# 3-Dimensional convolutional neural networks for predicting StarCraft Ⅱ results and extracting key game situations

**DOI:** 10.1371/journal.pone.0264550

**Published:** 2022-03-03

**Authors:** Insung Baek, Seoung Bum Kim

**Affiliations:** School of Industrial and Management Engineering, Korea University, Seoul, Republic of Korea; Kingston University, UNITED KINGDOM

## Abstract

In real-time strategy games, players collect resources, control various units, and create strategies to win. The creation of winning strategies requires accurately analyzing previous games; therefore, it is important to be able to identify the key situations that determined the outcomes of those games. However, previous studies have mainly focused on predicting game results. In this study, we propose a methodology to predict outcomes and to identify information about the turning points that determine outcomes in StarCraft Ⅱ, one of the most popular real-time strategy games. We used replay data from StarCraft Ⅱ that is similar to video data providing continuous multiple images. First, we trained a result prediction model using 3D-residual networks (3D-ResNet) and replay data to improve prediction performance by utilizing in-game spatiotemporal information. Second, we used gradient-weighted class activation mapping to extract information defining the key situations that significantly influenced the outcomes of the game. We then proved that the proposed method outperforms by comparing 2D-residual networks (2D-ResNet) using only one time-point information and 3D-ResNet with multiple time-point information. We verified the usefulness of our methodology on a 3D-ResNet with a gradient class activation map linked to a StarCraft Ⅱ replay dataset.

## Introduction

The gaming industry has grown to such an extent in popularity that one segment of it has been recognized as a new competition category, e-sports [1,2]. Various genres such as real-time strategy games, first-person shooter games (FPS), role-playing games (RPG), and multiplayer online battle arenas (MOBA) have proven especially popular. Real-time strategy games such as StarCraft, StarCraft Ⅱ, and MOBA games such as League of Legends and Dota 2 are leading the growth of the gaming industry. Numerous competitions related to these games are held and broadcast. Similarly, the number of people employed in e-sports, such as professional gamers, commentators, and coaching staff, continues to increase. In accompaniment with this growth, the problem of extracting key game situations has become an important research topic. Extracting key game situations means using in-game information to derive important situations based on forecasting the probability of a win or a loss. By addressing this issue, viewers of game broadcasts can satisfy their curiosity about a player’s winning or losing, and gamers can improve their skills and heighten their awareness of the importance of creating and employing their own strategies as well as apprehending those of their opponents.

However, accurately extracting key game situations can be challenging. The accurate extraction of key game situations requires an analysis of the interaction of in-game information, which is difficult because of both the sheer volume of information as well as the large number of ways it can be combined. For example, in StarCraft, information concerning units, buildings, and resources exists, but how this information is combined and presented hinges on a gamer’s actions [3]. At a certain point in time, gamers can choose one from a variety of options, including harvesting resources, attacking, building buildings, and scouting. Moreover, real-time strategy games like StarCraft are not limited to game playtime, making the total number of cases in one game almost infinite. The League of Legends poses the same difficulty. In it, each player chooses one champion from over the 100 champions available, and 10 champions can be played in any one game. Various situations occur, depending on the gold and other items collected by each champion, points for experience, and the players’ skill levels. Several battles are waged according to each champion’s and each team’s strategy, and the game results are determined through these battles. Because MOBA games like League of Legends are not time limited, the number of game situations is almost infinite.

Recently, several studies have applied machine learning and deep learning algorithms to various games. The machine learning and deep learning algorithms were tailored to find the relationship between various information in various games, including the StarCraft series [4–8]. Among the numerous win-loss predictive game studies, more have been conducted on the StarCraft series of games than on any other game. Several of those related to win-loss predictions used machine learning and deep learning [9–11]. Moreover, in StarCraft Ⅱ, many comparisons have been made of win-loss prediction models that used in-game information in conjunction with various machine learning and deep learning algorithms [12–15]. The StarCraft series, which has led the development of the gaming industry, has been played for more than 20 years. In this real-time strategy game, you can choose between the Terran, Protoss, and Zerg races, each of which has different characteristics, strengths, and weaknesses that act to create various game patterns [12]. The Terran comprise humans, the Protoss consist of alien humanoids with highly advanced technology, and the Zerg are creatures who use biological adaptation instead of technology [16]. Players gather two types of resources, minerals and gas, which they use to construct buildings and produce units. A player can win the game by destroying all enemy buildings and units or by the enemy player surrendering. The object in StarCraft Ⅱ is to collect resources, build structures, control various units, and create strategies for winning by predicting opponents’ behaviors, which are partially observable [3,15,17,18]. In this kind of game, it is important to construct a new strategy for each game based on a map, race, and presumed enemy strategy. To formulate a new winning strategy requires accurately analyzing the key situations that affect a game’s results.

While previous studies of StarCraft and StarCraft Ⅱ mainly focused on predicting the results of games, we propose a predictive model capable not only of predicting game results but also of identifying the key situations that influence those results. We used three-dimensional (3D) residual networks to construct a winning predictive model [19] and used gradient-weighted class activation mapping (Grad-CAM) to extract the key situation that affects the final game results [20–22].

The main contributions of this study can be summarized as follows:

1. In StarCraft Ⅱ, the game result is decided based on multiple time point battles rather than a single time point battle because the game is time dependent and each situation affects another situation. Therefore, we designed the 3D-ResNet model based on spatiotemporal information. Consequently, we proved that the model performance was improved by using multiple time points.
2. In the StarCraft Ⅱ game, winning or losing is decided by the interacting units and buildings in each pixel. Therefore, we adapted non-local neural blocks with 1×1 convolution filters that take into consideration all the input feature information. By taking advantage of these non-local blocks, the performance of the win-loss prediction model can be improved.
3. To analyze the key situation that affected the game result, Grad-CAM was applied. We could thus confirm the crucial part of the game having the most effect on the win-loss prediction.
4. In research investing StarCraft Ⅱ, this study was the first time in which the image form of game replay was used as input data for a deep learning model. Because we used images as input data, we could extract important situations more intuitively.

The remainder of the paper is organized as follows. The next section consists of a review of the studies on StarCraft and StarCraft Ⅱ. The third section illustrates the details of the proposed win-loss prediction model and the methodology of extraction of the key game situations, while the 4^th^ Section presents the qualitative and quantitative experimental results. Finally, Section 5^th^ contains our concluding remarks and directions for future research.

## Related works

Several win-loss predictive game studies have applied machine learning and deep learning algorithms to various games. Semenov et al. compared Dota2, a MOBA game, with several machine learning models including naïve Bayes, logistic regression, factorization machines, and an XGBoost [7]. Of these, the factorization machines performed the best. Kang and Lee presented a win-loss probability model using a multilayer perceptron model (MLP) for the League of Legends game [8]. They secured a good performing win-loss probability model by using the image and text data from a YouTube video as the input.

Some studies of StarCraft have used machine learning models that use various information as input data to predict winners and losers. Erickson and Buro made win-loss predictions by using a logistic regression model with 400 “Protoss vs. Protoss” game replay data. They extracted from StarCraft replay data such features as resources, buildings, units, the estimated value of players’ control skills, and map information [9]. For predicting the game winner, they used a logistic regression model. However, prediction accuracy with this model did not exceed 75%. Later, Ravari et al. suggested predicting game results by using ensemble models such as the random forest and gradient boosting machine models [10]. They used information separated by time-dependent and time-independent features to evaluate the performance of each model according to the time-dependent features. Overall, their model performed well when time-dependent features and action per minute (APM) information were used. After designing the random forest and the gradient boosting machine using those features, they extracted important features based on scores calculated by the model. However, its accuracy in predicting winners did not exceed 70%. Moreover, because the model could only extract in-game components such as income, resources unspent, and micro commands, it was limited in its capability to identify the key situations of games. Unlike other StarCraft win-loss prediction studies, Álvarez-Caballero et al. compared six algorithms—naïve Bayes, logistic regression, k-nearest neighbor (KNN), neural networks, random forest, and gradient boosting tree—in terms of good predictive performance early in a game [11]. With the KNN model, they achieved good performance with only the first 10 minutes of game data. However, the KNN model, like the others, fell short in its capability to identify the key points that determined the outcome of games. Several win-loss prediction studies used in-game extracted information in a real-time strategy game that collected resources, built buildings, and produced units. However, the difference between previous studies and the present study was the form of the input data. In this study, we extracted image form as input data. In addition, although previous studies found it challenging to extract important situations, this study differs in that it is possible to extract important situations through image data analysis.

StarCraft Ⅱ has also been the subject of several studies that have attempted with varying degrees of success to use in-game information to predict winners or losers. Leblanc and Luis compared random forest, AdaBoost, and naïve Bayes models in terms of their predictive accuracy and found that the naïve Bayes model performed best [12]. They used units, buildings, APM, and control-related information as the model’s inputs. They also experimented with a setting of two criteria. First, they divided the in-game information into temporal features and basic features to confirm the temporal information effect that consists of information combining previous time features with basic features. Second, they defined how to use each player’s information. They achieved their best performance from the model that used temporal features and difference information between two players. Wu et al. proposed predicting game outcomes by using recurrent neural networks (RNN) with gated recurrent unit (GRU) cells in the last two layers [13]. The RNN and GRU use time-series information for predictions. However, as in other previous studies, the focus was on game outcomes without attempting to explain why the results occurred. In still another approach, Lin et al. suggested relying on dividing the feature information in StarCraft Ⅱ based on whether it related to the game player or to their enemy [14]. First, non-adversarial features defined the information, such as the resources collected and spent by the player, the number of units produced, and the percentage of time devoted to the upgrade. Second, the adversarial features were defined as content related to enemy information, such as the number of enemy units killed, destroyed structures, and the proportions of the enemy units. They compared the performance of the neural processes and support vector machine models based on the aforementioned non-adversarial and adversarial features. Ultimately, a neural processes model using only the adversarial features performed best in predicting winners and losers, achieving over 80% accuracy despite using a training size of only 200 data points. A study by Lee et al. proposed a combat winner prediction model considering unit combination and battlefield information [15]. In researching StarCraft Ⅱ, most previous studies used summarized information, not image form data; therefore, these studies did not extract key situations intuitively. In StarCraft Ⅱ, as in StarCraft, researchers have pursued excellence in results prediction, but none of their results have been satisfactory in pinpointing and extracting information on those key game situations that determine who wins and who loses.

Other studies have been attempted to train StarCraft agents with high game win rates based on reinforcement learning algorithms [23–25]. For example, AlphaStar, a StarCraft Ⅱ agent, used a variety of methods, such as neural network architectures, imitation learning, reinforcement learning, and multi-agent learning. The neural network architecture was used to learn StarCraft Ⅱ replay image data. Vinyals et al. applied imitation learning to effectively learn a large number of human demonstrations [23]. They proposed a new multi-agent training system with different exploiters whose purpose is to strengthen the main agents. AlphaStar, in the full game of StarCraft II, was evaluated through a series of online games against human players. AlphaStar was rated at Grandmaster level and above 99.8% of officially ranked human players for StarCraft. It focused on creating an agent that can play StarCraft games well. Lee et al. [24] presented a novel modular architecture, which splits responsibilities between multiple modules in StarCraft II. Each module controls one side of the game, and two modules are trained with self-play. They achieved 92%-win rates against the built-in bot in Zerg vs. Zerg matches. Pang et al. trained a StarCraft Ⅱ agent through the two levels of hierarchical policy training techniques of reinforcement learning for full-length games in StarCraft [25]. One is the macro-actions extracted from demonstration trajectories of experts, which can reduce the action space yet remain effective. The other is a two-layer architecture, which is modular and easy to scale. As a result, they could achieve over 93% winning rate against the most challenging built-in AI (level-7). The studies based on reinforcement learning focus on creating a high-performance StarCraft Ⅱ agent, but the present study differs in that it predicts the outcome of a game and extracts the key situations based on information played by real people.

## Proposed win-loss prediction model

### Win-loss prediction model

In this study, we used StarCraft Ⅱ replay data as the input. Because all StarCraft Ⅱ replay data differs in length, we sampled 50 time points per replay to efficiently train a deep learning model. These 50 time points are a hyperparameter that a researcher can set. In the Results section, we also included the experimental results according to the number of sampling frames. Although we sampled 50 frames, as the model training epochs increase, the model was trained by looking at most of the frames in the entire replay data because, when training the model, it extracted different frames from the same replay data every epoch. For example, 50 frames are extracted in the first epoch, including the 15th, 30th, and 100th. Fifty frames are again extracted in the second epoch, but they now include the 19th, 40th, and 90th. Sampling weights were used as log weights to do more sampling near the last time points that contained a large amount of information related to the game’s result. Furthermore, to address a sparsity problem caused by the binarization of categorical variables, we transformed categorical variables into continuous variables using the embedding layer. Consequently, we acquired the 3D tensor with 120×128×128 per time point. After all the preprocessing was completed, we used 3D-ResNet to design a model to predict winners. The 3D-ResNet model is based on the residual network (ResNet) architecture [26].

**Fig 1:**
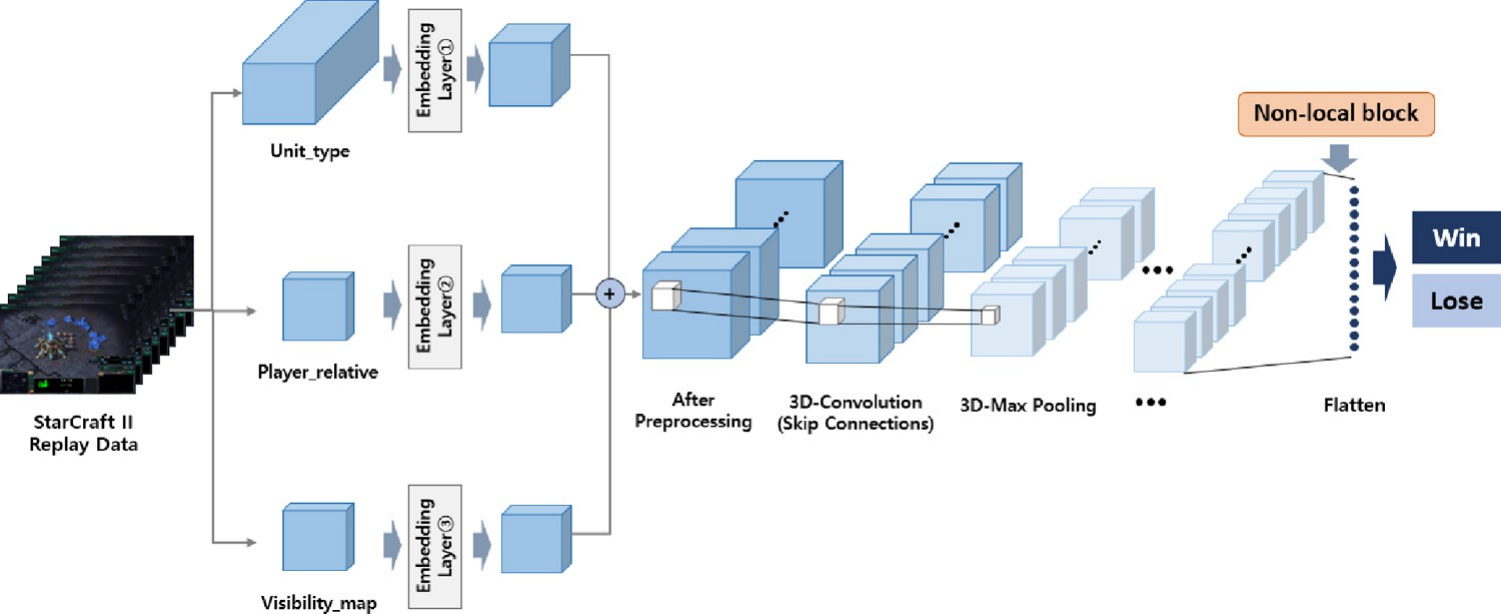
3D-ResNet model with non-local neural blocks.

Resnet architecture placed first in the 2015 ImageNet Large Scale Visual Recognition Competition (ILSVRC) by addressing the performance degradation problem in deeper neural networks [26]. The formulation of *F*(*x*)+*x* can be implemented by neural networks with shortcut connections that can skip a few layers. Shortcut connections perform mapping, and their outputs are added to those of the stacked layers [27]. Deeper neural network training has been improved by residual learning based on these short connections. After designing the 3D-ResNet model, we inserted a non-local neural block after the last convolutional layer to achieve higher model performance. We determined the best non-local neural block location through several attempts at a comparative experiment. Finally, we designed the winner prediction model architecture, as shown in Fig 1. As shown in Fig 1, all parameters in a deep learning model were trainable, except for the embedding and pooling layers.

A non-local block captures long-range dependency in space and time to deal with occlusion and misalignment [28]. In accordance with the non-local mean operation [29], we defined a general non-local operation in various deep neural networks where *i* is the index of an output position (in space, time, or spacetime) and *j* is the index that enumerates all possible positions computed by the matching *i* index [30]. *x* is the input data (e.g., image, video), and *y* is the output data of the same size as *x*. A pairwise function *f* computes a scalar between *i* and all *j*, while the function of *g* computes a representation in an embedded space of the input data at position *j* with weight matrix *W*_*g*_. The normalization factor is set as *C*(*x*) = ∑_∀*j*_ *f*(*x*_*i*_, *x*_*j*_)

$$y_{i}=\frac{1}{C(x)}\sum_{\forall j}f(x_{i},x_{j})g(x_{j}),$$ (1)

$$g(x_{j})=W_{g}x_{j}.$$ (2)

Wang et al. proposed several types of *f* function; however, the non-local block is not sensitive to these choices [30]. The following four functions are the typical *f* choices:

$$Gaussian:\;f(x_{i},x_{j})=e^{{x_{i}}^{T}x_{j}},$$ (3)

$$Embedded\;Gaussian:\;f(x_{i},x_{j})=e^{\theta ({x_{i})}^{T}\varphi (x_{j})},$$ (4)

$$Dot\;product:\;f(x_{i},x_{j})=\theta {\left(x_{i}\right)}^{T}\varphi (x_{j}),$$ (5)

$$Concatenation:\;f(x_{i},x_{j})=ReLU(w_{f}^{T}[\theta (x_{i}),\varphi (x_{j})]),$$ (6)

$${where\;\theta (x}_{i})=W_{\theta }x_{i}\;and\;\varphi (x_{j})=W_{\varphi }x_{j.}$$

Then the non-local block is defined by using the residual connection based on Eq 1:

$$z_{i}=W_{z}y_{i}+x_{i}.$$ (7)

where *y*_*i*_ is given in Eq 1, and +*x*_*i*_ means a residual connection. In Eqs 6 and 7, *W*_*f*_, *W*_*θ*_, *W*_*φ*_, and *W*_*z*_ represent the weight matrices. Thus, in the non-local block modules, all weight matrices are trainable parameters. A non-local block is very flexible and can be easily inserted into any existing architecture [30].

**Fig 2:**
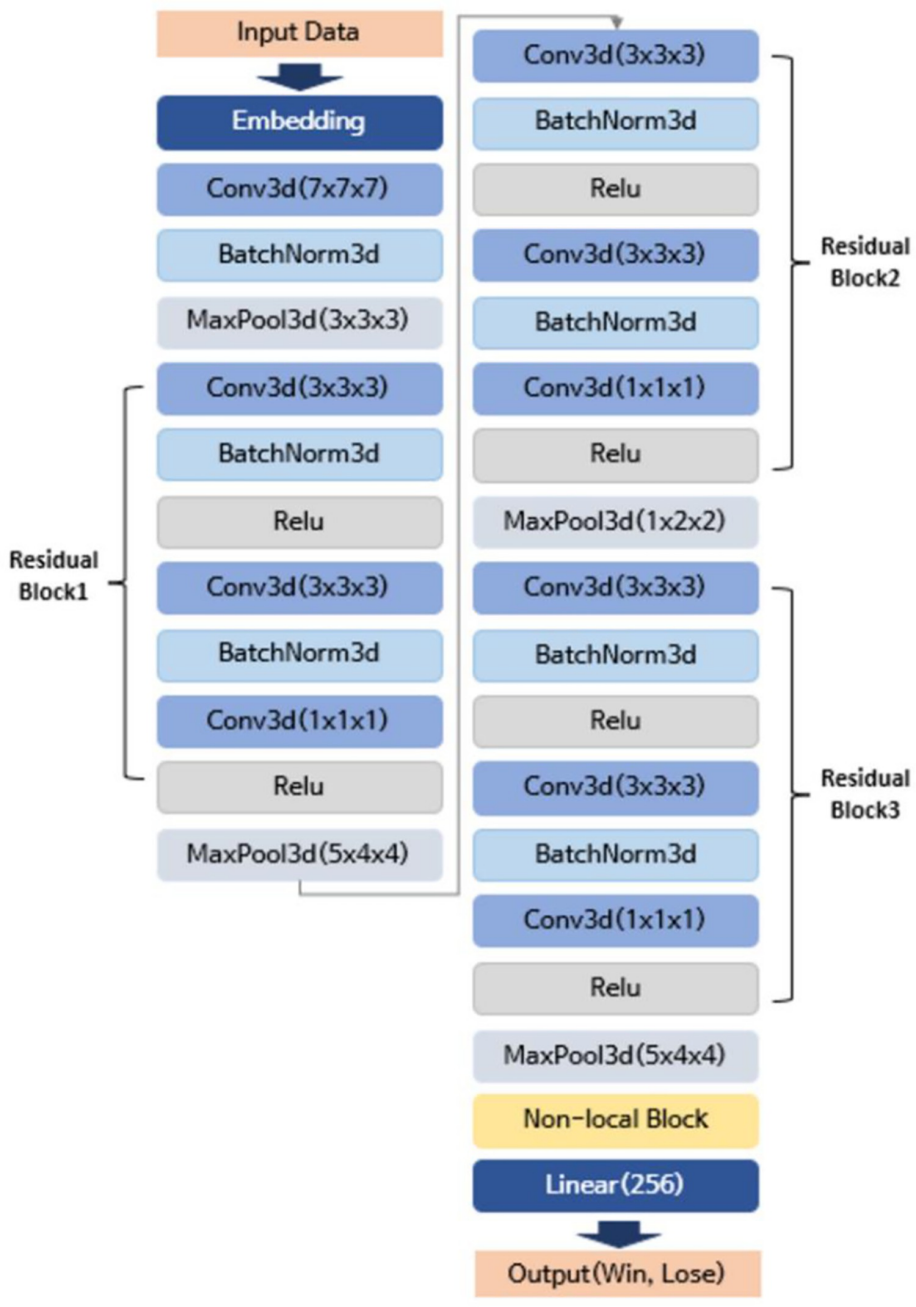
Architecture of the 3D-ResNet with non-local neural blocks.

Fig 2 shows the detailed architecture of the 3D-ResNet model with non-local neural blocks. The model is trained on the training set per each batch. The learning rate is 0.001 with an adaptive moment estimation (Adam) and a batch size of four. The optimization algorithm uses a binary cross-entropy loss (BCE loss) function in which *y* is a binary indicator and *p* is the predicted probability of a certain class.


BCEloss=−(ylog(p)+(1−y)log(1−p)). (8)

## Extraction of key situations

**Fig 3:**
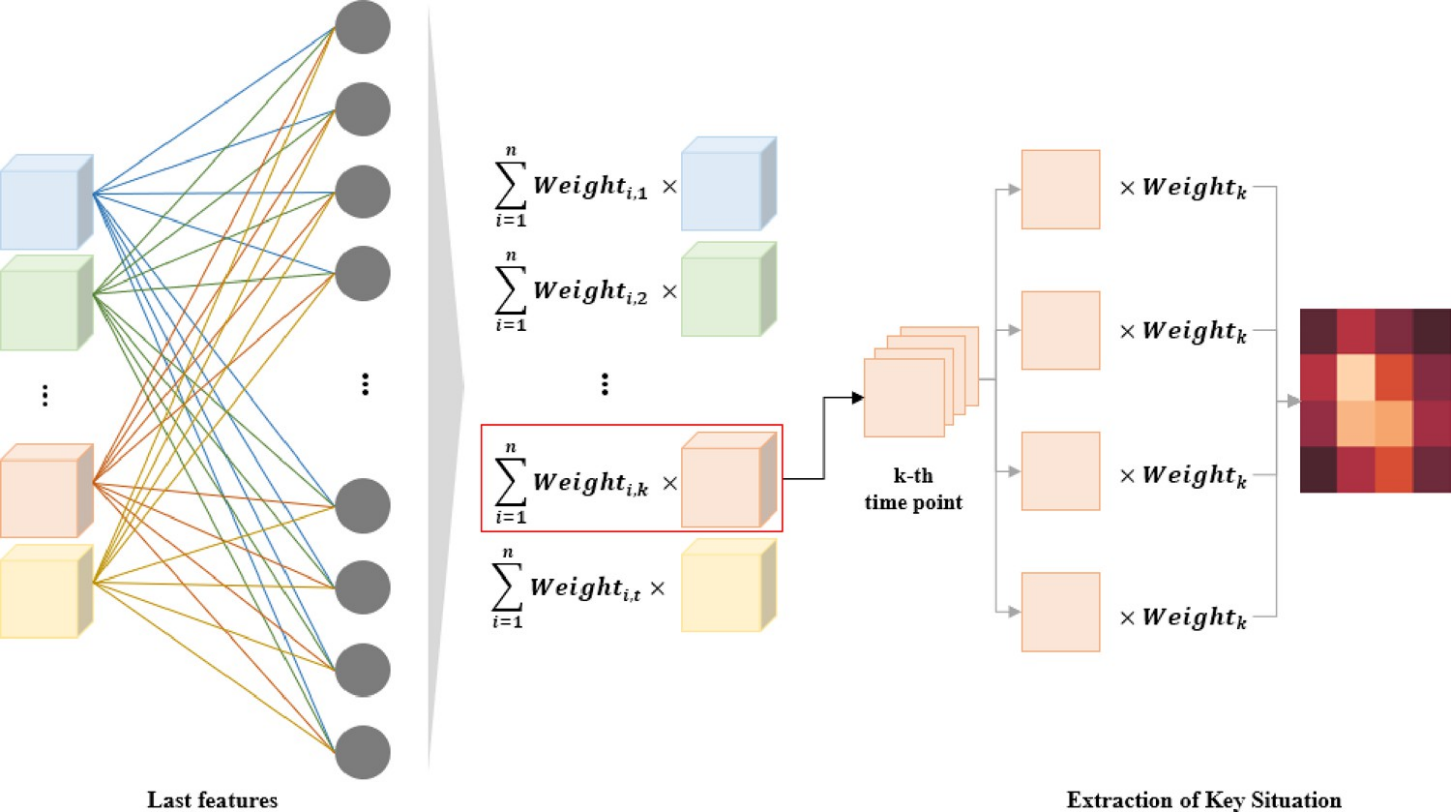
The Grad-CAM procedure in the extraction of a key situation at a certain time point in StarCraft Ⅱ.

After designing a model to predict winners, Grad-CAM was used to extract the key situations in a game. The Grad-CAM weights use gradients computed with the last layer’s weights in the trained model, i.e., we used the last gradient score in the completed training model. We obtained the key situations at a specific time point related to the game result through the last feature map multiplied by the Grad-CAM weights. The bright color in a pixel signifies the most important key factor at that time point. Fig 3 shows how Grad-CAM was applied in this study.

Grad-CAM is a technique for producing “visual explanations” for prediction results in CNN-based models [31]. It uses gradient information flowing into the last convolutional layer of the CNN to determine the pixels important to a decision of interest [22]. To obtain a class discriminative localization map for any class ***c***, the method first computes the following gradient of the score *y*^*c*^ (before Softmax) with respect to feature maps *A*^*k*^ of the last convolutional layer:

$$g_{c}(A^{k})=\frac{\partial y^{c}}{\partial A^{k}}.$$ (9)

where *k* indicates the channel index. The method then computes the following importance of weight $\alpha_{k}^{c}$ in each feature map *k* for a target class *c*:

$$\alpha_{k}^{c}=\frac{1}{Z}\sum_{i}\sum_{j}\frac{\partial y^{c}}{\partial A_{i,j}^{k}}.$$ (10)

where *Z* is the sum of all pixel values in feature map *k*, and (*i*,*j*) is the spatial index. The weight $\alpha_{k}^{c}$ represents the gradient weight. Finally, we obtained the following Grad-CAM, which is a weighted sum of feature maps using a rectified linear unit (ReLU) operator:

$$L_{Grad-CAM}^{c}=ReLU\left(\sum_{k}\alpha_{k}^{c}A^{k}\right).$$ (11)

Grad-CAM visualizes based on a heat map, converting the same size of the original image into the size of a specific class *c*.

## Experiments

### Data

**Fig 4:**
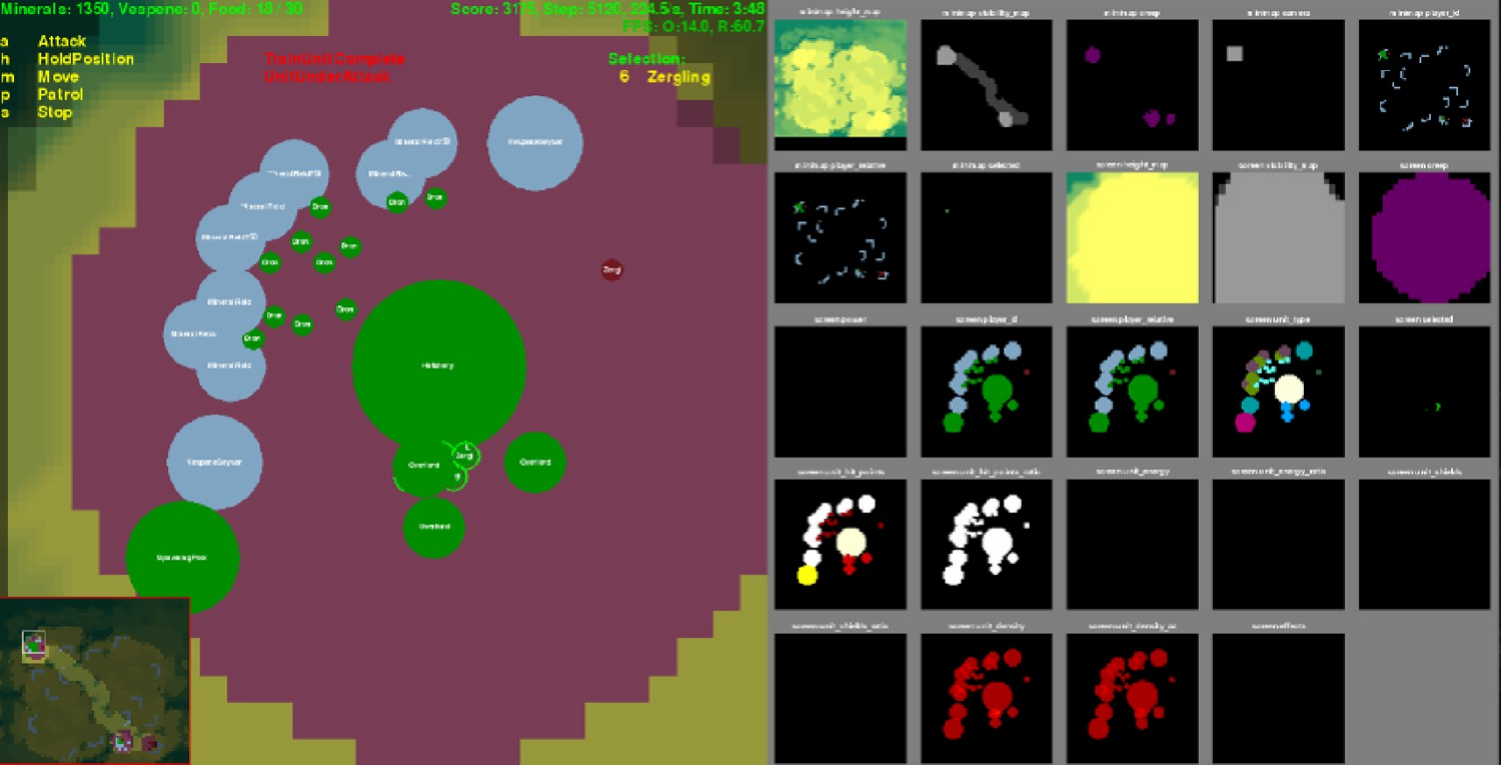
Parsed StarCraft Ⅱ features using the SC2LE.

We collected StarCraft Ⅱ replay data as played by game users on Battle.net. Variables were extracted using the StarCraft Ⅱ learning environment (SC2LE) package developed by DeepMind, which can be used to extract various game information from StarCraft Ⅱ replay data (https://github.com/deepmind/pysc2). Fig 4 shows the StarCraft Ⅱ features parsed by using the SC2LE package. The extracted information included player_relative, visibility_map, and unit_type, all of which are categorical data. Player_relative determines whether the unit is an enemy or a friendly army, visibility_map is related to the map’s field of view, and unit_type contains information such as units, resources, and buildings. In this study, we obtained 1,725 Terran versus Protoss replays. Of these, 1,227 were used as training data, and 498 were used as testing data. Based on the training data, the proportion of games won by Protoss was 53.5%, implying that the results were not biased in favor of one side or the other. The source code and data of the proposed method are available via https://github.com/InsungBaek/StarCraft2.

## Results

We conducted experiments with a win-loss prediction model in several scenarios to achieve the best performance. The model’s performance was evaluated by accuracy, recall, precision, and F1-score from the testing data. Table 1 shows a confusion matrix, typically used in binary classification problems (here win or lose). In this study, the games won by the Protoss are the accuracy criterion for the ratio of correctly predicted winning games in all data, recall is the ratio of correctly predicted Protoss winning games among all games actually won by Protoss, and precision is the ratio of games actually won by Protoss among all predicted Protoss victories. The F1-score can be computed by a harmonic mean of recall and precision.


$$Accuracy=\frac{n^{11}+n^{22}}{n^{11}+n^{12}+n^{21}+n^{22}},$$ (12)

$$Recall=\frac{n^{11}}{n^{11}+n^{12}},$$ (13)

$$Precision=\frac{n^{11}}{n^{11}+n^{21}},$$ (14)

$$F1‐Score=2\times \frac{Precision\times Recall}{Precision+Recall}.$$ (15)

**Table 1:** The confusion matrix of predicted the game results.

	Predicted Win	Predicted Loss
Actual Win	*n* ^11^	*n* ^12^
Actual Loss	*n* ^21^	*n* ^22^

We repeated 50 experiments to guarantee the general model performance because the test data changed with every time sampling. We compared the win-loss prediction model using only one time-point information and the win-loss prediction model using multiple time-points information to prove that the proposed model outperforms. Table 2 shows a comparison of the performance of the 2D-residual networks (2D-ResNet) and the 3D-ResNet models according to game time length. The 2D-ResNet model predicts wins or losses by using only one time-point information, and the 3D-ResNet model predicts wins or losses by utilizing the multiple time-points information in the game. After splitting the game time into four parts in 25% increments, we compared the performance of the 2D-ResNet and 3D-ResNet models to check if there was any difference between their performance during specific sections of the game. In Table 2, we name the first quarter, second quarter, third quarter, fourth quarter, and full length in the game length column. For example, in a 20-minute game, the first quarter is 0–5 minutes, the second is 5–10 minutes, the third is 10–15 minutes, and the fourth is 15–20 minutes. The full length represents 0–20 minutes, i.e., the complete game. The results confirmed that the test accuracy of the 3D-ResNet model was higher than that of the 2D-ResNet model in all parts except the first quarter. In more detail, the 2D-ResNet and 3D-Resnet models both showed performance that did not exceed 60% accuracy in either the first or the second quarter because the actual game win or loss was affected by a situation occurring after those parts of the game. After the halfway point, which is an important time for winning or losing the game, we confirmed a relatively large difference between the 2D-ResNet and 3D-ResNet models. In particular, in the fourth quarter, the 3D-ResNet model showed about a 10% higher performance than the 2D-ResNet model. Finally, when using the time information for the entire game, the 3D-ResNet model performance was better. When comparing the 2D-ResNet and 3D-Resnet models, we used one time point in 2D-ResNet and 50 time points in 3D-ResNet after sampling 50 time points in a specific interval.

**Table 2:** The winning prediction model’s performance concerning game time length.

Game Length	Model	Test Accuracy	Test Recall	Test Precision	Test F1-Score
First quarter	2D-ResNet	**0.562**	0.562	**1.000**	**0.720**
	3D-ResNet	0.558	**0.603**	0.622	0.613
Second quarter	2D-ResNet	0.586	0.592	**0.852**	**0.698**
	3D-ResNet	**0.591**	**0.601**	0.806	0.689
Third quarter	2D-ResNet	0.602	0.600	**0.873**	**0.712**
	3D-ResNet	**0.633**	**0.639**	0.800	0.710
Fourth quarter	2D-ResNet	0.786	0.789	0.844	0.816
	3D-ResNet	**0.888**	**0.910**	**0.887**	**0.899**
Full Length	2D-ResNet	0.617	0.622	0.813	0.705
	3D-ResNet	**0.880**	**0.879**	**0.913**	**0.895**

Table 3 shows the results when using non-local blocks with different *f* functions. Wang et al. proposed non-local blocks with four *f* functions: Gaussian, dot-product, concatenation, and Gaussian embedded [30]. However, different performances occurred depending on which functions were chosen in solving a task. Hence, we applied the four functions in non-local blocks after the last convolutional layer and compared them with all results. We achieved the highest test accuracy, 88.6%, when using the Gaussian embedded function.

**Table 3:** The winning prediction model’s performance with different *f* functions in non-local blocks.

Model	Function *f*	Test Accuracy	Test Recall	Test Precision	Test F1-Score
3D-ResNet	-	0.880	0.879	0.913	0.895
3D-ResNet with non-local block	Gaussian	0.853	0.859	0.884	0.871
	Dot-product	0.867	0.892	0.869	0.880
	Concatenation	0.877	**0.901**	0.877	0.889
	**Gaussian, embeded**	**0.886**	0.880	**0.924**	**0.901**

In addition, we performed experiments depending on the location and count of non-local blocks. Table 4 shows the results on non-local block location. Because we used the ResNet-18 architecture, the non-local blocks are located in three cases: after the first residual block, after the mid-residual block, and after the last residual block. We conducted experiments in only two cases after the mid-residual block and after the last residual block because of the GPU (NVIDIA GeForce RTX 2080 Ti) memory voltage. In Table 4, residual block2 signifies after the mid-residual block, and residual block3 represents after the last residual block. Consequently, we achieved the highest model performance at a non-local block located after the last residual block.

**Table 4:** The winning prediction model’s performance with different locations of each non-local block.

Model	Location	Test Accuracy	Test Recall	Test Precision	Test F1-Score
3D-ResNet	-	0.880	0.879	0.913	0.895
3D-ResNet with non-local block	Residual block2	0.880	**0.889**	0.899	0.894
	Residual block2 and Residual block3	0.866	0.870	0.895	0.882
	**Residual block3**	**0.886**	0.880	**0.924**	**0.901**

We conducted a comparative experiment with machine learning models including logistic regression, random forest, XGBoost, light GBM models, and other CNN architectures to prove the validity of the proposed 3D-ResNet structure. Most previous StarCraft II prediction studies used machine learning models using the summarized values for each game frame. In addition, we compared different CNN architectures such as 3D-AlexNet and 3D-VGGNet. Table 5 shows that the proposed 3D-ResNet models outperformed other CNN architectures and machine learning models. The 3D-AlexNet model, consisting of five convolutional layers and three fully connected (FC) layers, used ReLU activation functions to improve its performance [32]. The 3D-VGGNet model increased depth by using an architecture with 3×3 convolutional filters to improve the model’s performance [33]. We used a 0.0001 learning rate with an Adam optimizer considering weight decay (AdamW) in the 3D-AlexNet and 3D-VGGNet models. Except for the optimizer and learning rate, other hyperparameters were all the same as the proposed experiment settings. The 3D-VGGNet model consisted of seven convolutional layers and three FC layers to suit our data.

**Table 5:** The winning prediction model’s performance with different machine learning and deep learning models.

Model	Test Accuracy	Test Recall	Test Precision	Test F1-Score
Logistic Regression	0.517	0.527	0.453	0.487
Random Forest	0.490	0.659	0.443	0.529
XGBoost	0.511	0.530	0.449	0.486
Light GBM	0.501	0.596	0.446	0.509
3D-AlexNet	0.675	0.719	0.692	0.705
3D-VGGNet	0.843	0.845	0.883	0.863
**3D-ResNet**	**0.886**	**0.880**	**0.924**	**0.901**

**Fig 5:**
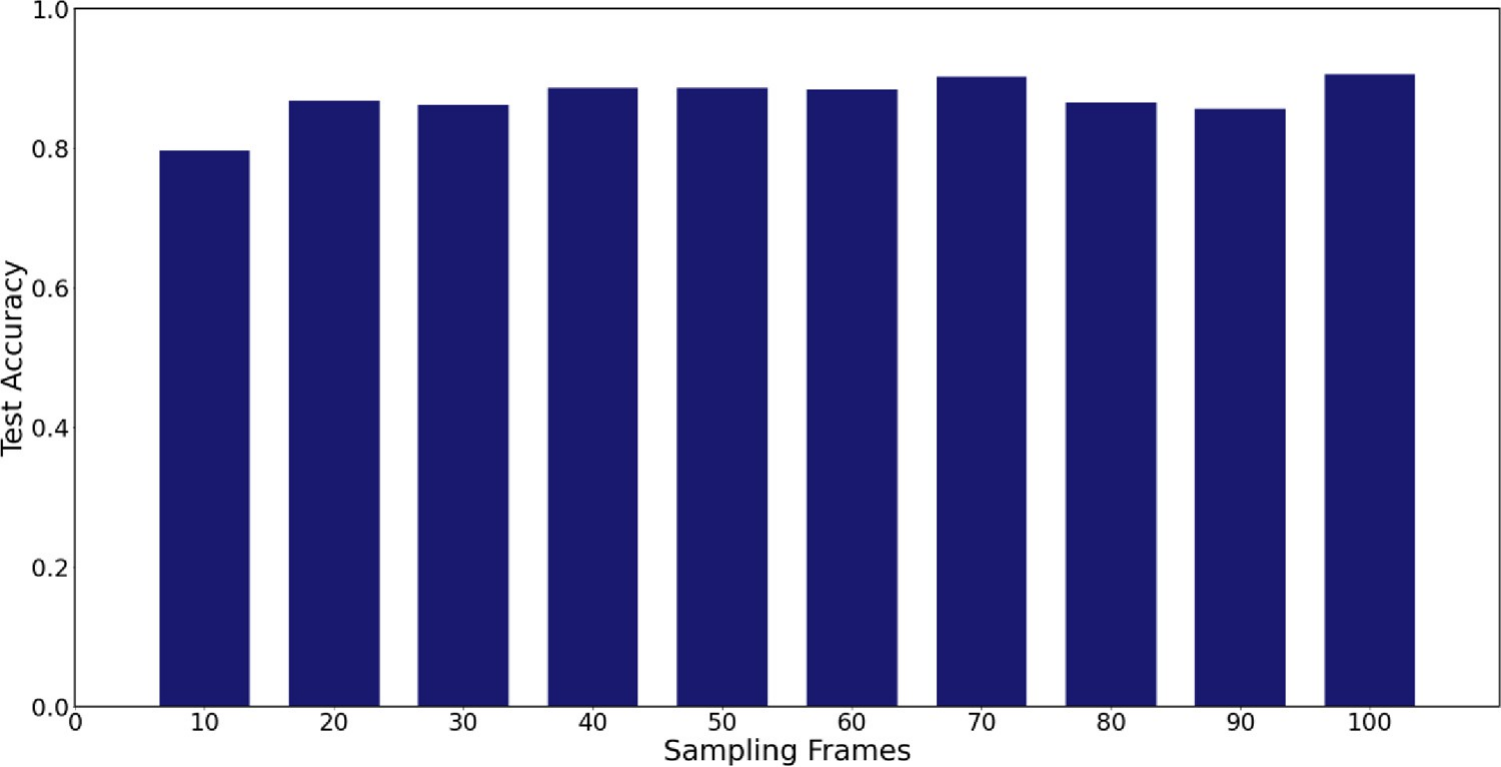
Win-loss prediction model performance with different number of sampling frames.

To see how the performance of the model changes with the number of sampling frames, we examined testing accuracies by changing the number of sampling frames from 10 to 100 at 10 intervals. Fig 5 shows all have a higher than 85% performance, except for the 10-sampling frame sampling, implying that the model’s performance was not significantly affected by the number of sampling frames. It is worth noting that when using 100-sampling frames, the model’s performance tended to increase. However, it requires twice as much time as 50-sampling frames. Therefore, in this study, we used 50-sampling frames for good performance as well as for efficiency.

**Fig 6:**
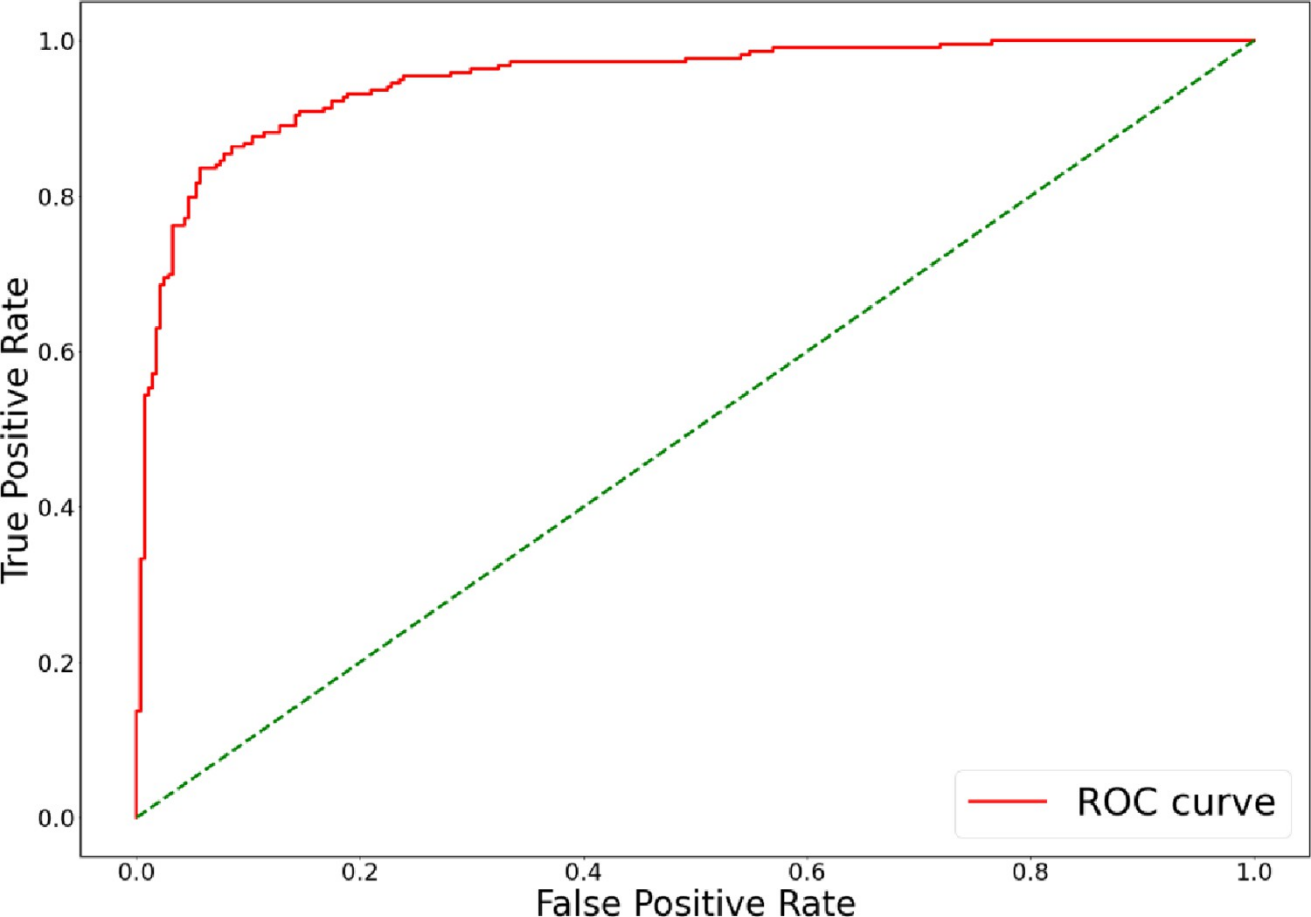
The ROC curve in our proposed model.

Moreover, we confirmed the superiority of the proposed method based on the receiving operator characteristics (ROC) curve that measures the performance of a classification model consisting of the true positive rate (TPR) and false positive rate (FPR). We could evaluate the performance of the model through the area under the ROC curve (AUC). Fig 6 shows the ROC curve of our proposed model; the AUC is 0.95, demonstrating the superiority of the proposed model.

**Fig 7:**
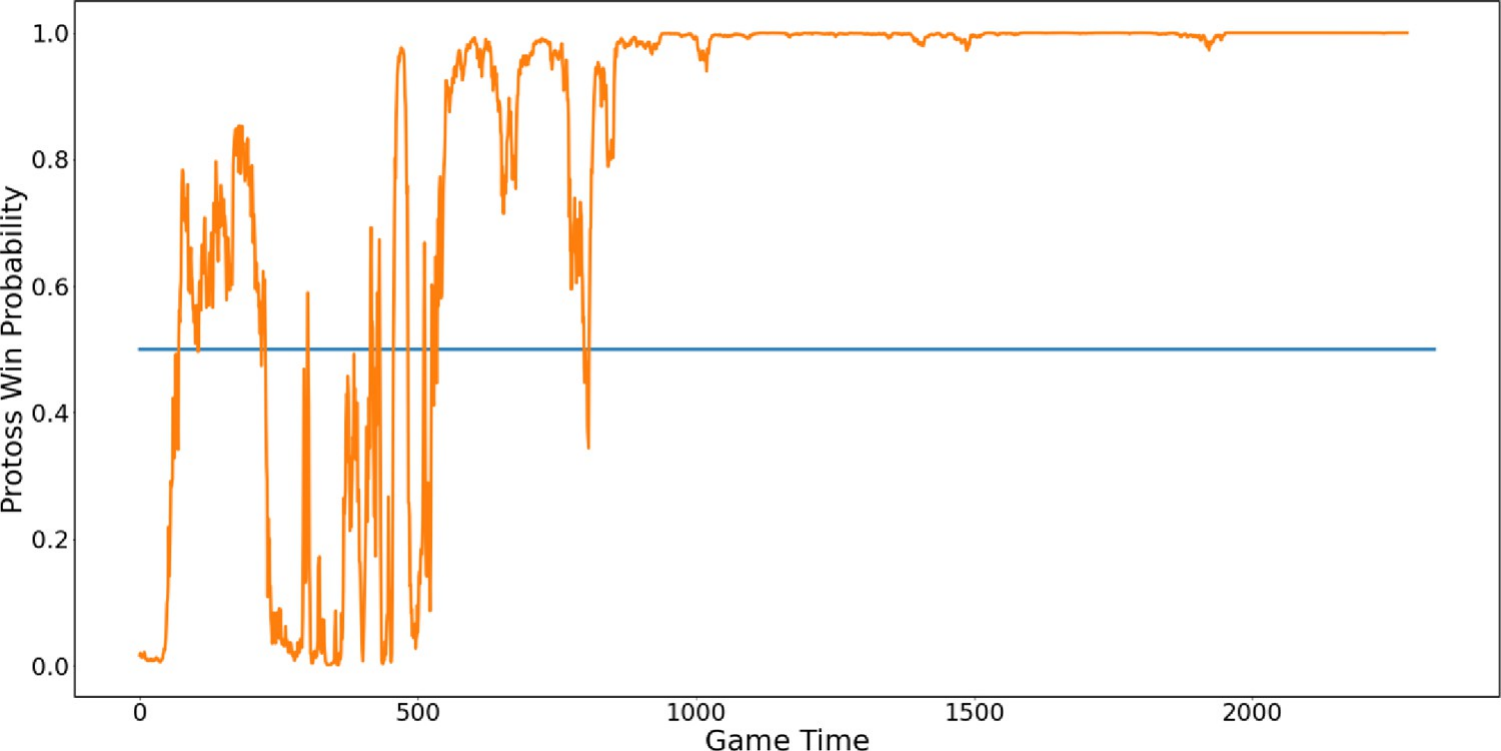
Protoss win probability per frame using a sliding window.

Finally, to see the prediction probability over time, we calculated a continuous winning probability in the game. In Fig 7, we show an example of a game won by the Protoss; a win probability value closer to one indicates who won the game. At the beginning of the game, when we are unsure who will win, the probability of the Protoss winning fluctuates significantly. However, after a few battles, we identified the stable probability of the Protoss winning from mid-game on.

**Fig 8:**
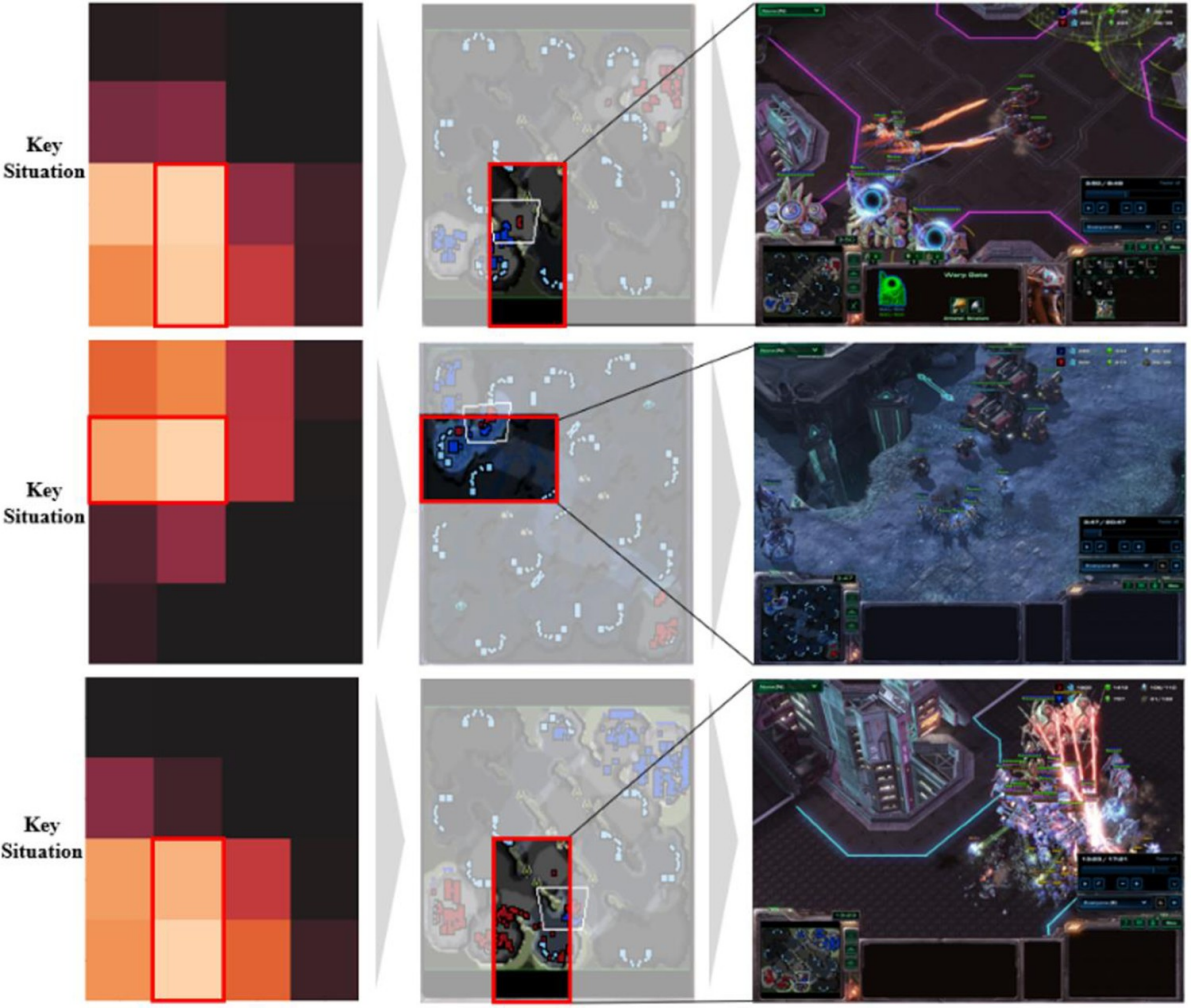
Example of the Protoss winning games: Grad-CAM focused on the scene in which Protoss defeated Terran in combat.

**Fig 9:**
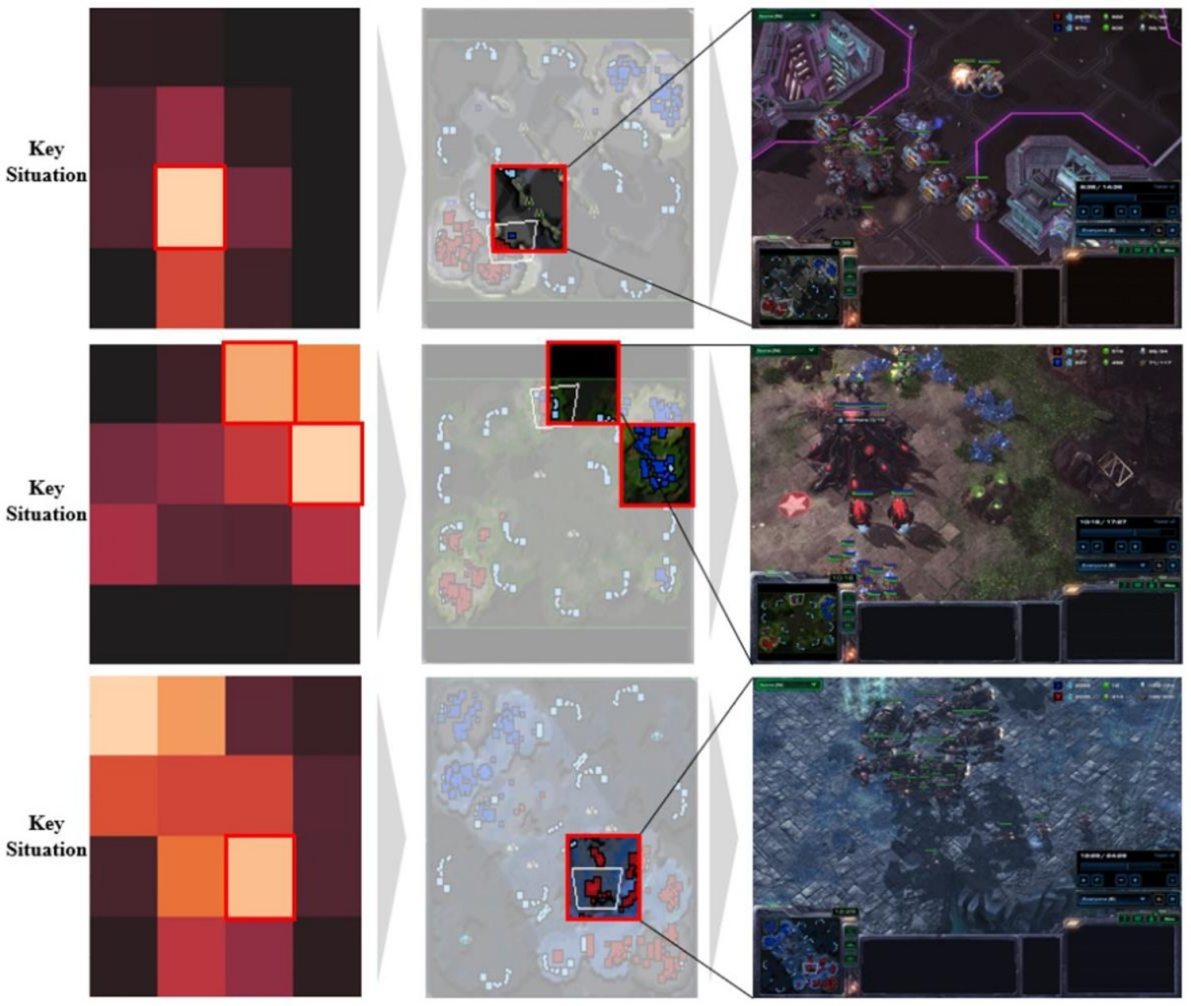
Example of the Terran winning games: Grad-CAM focused on the scene in which Terran defeated Protoss in combat.

The accuracy of key situations could be evaluated qualitatively through intuitive examples, which are shown in Figs 8 and 9. Fig 8 is an example of the Protoss winning games. The critical situations were in the scene in which the Protoss defeated the Terran in combat. The first of the three examples in Fig 8 shows that the Protoss were well protected from the Terran’s attacks. Afterwards, the Protoss were able to gather resources and gather units to defeat the Terran smoothly. In the case of the example in the middle, it was a game in which Protoss attacked Terran early and kept their advantage until the end of the game. The last example shows that Protoss won a big battle with the Terran units and could easily win the game. Fig 9 is an example of the Terran’s winning games. The key situations were in the scene in which the Terran defeated the Protoss in combat. Of the three examples in Fig 9, the first case shows that Terran was well defended against Protoss’s attacks. After that scene, Terran won the game by harvesting a large amount of resources and producing many units. In the second example in Fig 9, the Terran attacked the Protoss resource-harvesting area to create an advantage, while the last example in Fig 9 shows that Terran easily gathered a large number of battlecruisers to attack Protoss. In both instances, we can see that the scene which had the greatest influence on who won the game was extracted.

**Fig 10:**
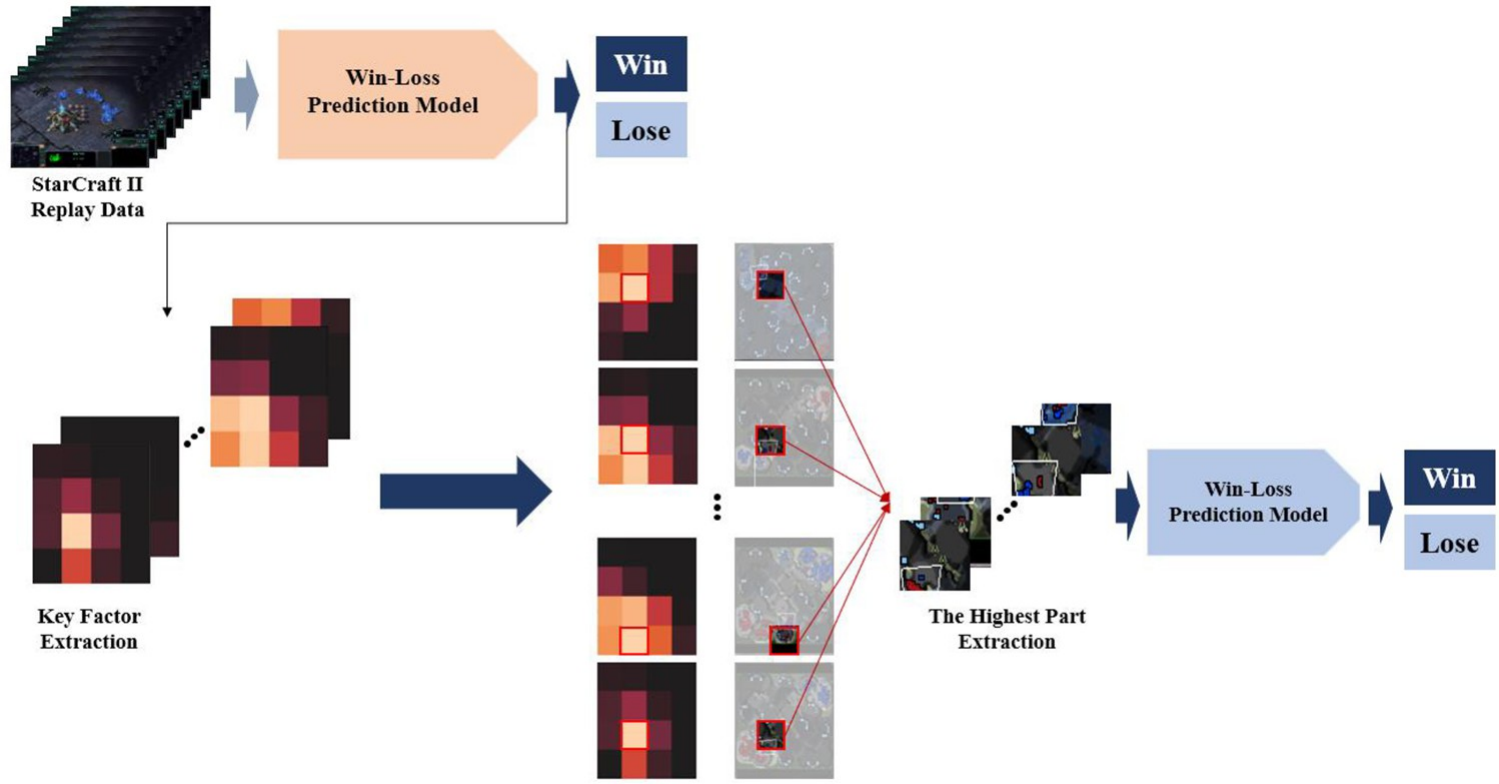
The process of Grad-CAM evaluation based on the highest Grad-CAM score.

Fig 10 shows our attempt to evaluate the effectiveness of Grad-CAM numerically. After extracting important situations from the pre-trained win-lose prediction model, we extracted the part with the highest Grad-CAM score at each time point. Then, after cropping the area that matches the part from the original data, we trained a new win-loss prediction model with the corresponding area. Similarly, we proceeded with the lowest Grad-CAM score.

Finally, we experimented with a randomly cropped part of the original replay data. Each experiment was repeated five times to measure the results. As shown in Table 6, the model performance was high when using the part with the highest Grad-CAM score, showing that it had effectively extracted the meaningful situation for the game results through the proposed methodology. We also confirmed that the model performance when using the lowest Grad-CAM score was better than when using the random area. Because this problem is a binary classification one, we guessed that the area of the high Grad-CAM score affected the prediction of the game’s winner. In contrast, we guessed that the part with the lowest Grad-CAM score affected predicting the game’s loser. Therefore, in both cases, the prediction performance was better than the randomly cropped region. We then confirmed that the part predicted with the area of the highest Grad-CAM score had the best model performance. In conclusion, we determined which scene was the turning point in the game and was the key scene to extract. Ravari et al. used feature importance scores in random forest and gradient boosting machine models to identify important features [10]. However, their important features were limited to the extraction of in-game components such as income, resources unspent, and micro-commands. It seems that their proposal was not intended to analyze critical game situations. In our study, however, the proposed method is used to analyze and extract the key situation critical to a game’s outcome.

**Table 6:** Comparison of model performance based on the area corresponding to the Grad-CAM. score.

	The highest Grad-CAM score	The lowest Grad-CAM score	Random
Test Accuracy	**0.900**	0.858	0.698

## Conclusions

In this study, we proposed a methodology to extract key situations in real-time strategy games such as StarCraft Ⅱ. First, we designed a model to predict winners with an accuracy close to 0.9. Then, we used Grad-CAM to extract key situations that determined the outcomes of games. When we confirmed the original replay data, we could ascertain those key factors. Using the proposed methodology, we were able to analyze game results accurately and devise various ways to win. If the results of this study were applied in the real world of gamers, it would promote the development of the gaming industry and improve the performance of gamers and the quality of game broadcasts. However, in cases in which the game results were not accurately predicted, we were unable to ascertain the key factors in the game outcomes. This shows the need for more accuracy in our predictive model; hence, in our future work we will be parsing more variables related to real-time strategy games, such as control and strategy development.
